# Comparative In Vitro Cytotoxicity Study of Carbon Dot-Based Organometallic Nanoconjugates: Exploration of Their Cell Proliferation, Uptake, and Localization in Cancerous and Normal Cells

**DOI:** 10.1155/2022/3483073

**Published:** 2022-03-15

**Authors:** Eepsita Priyadarshini, Ramovatar Meena, Himadri B. Bohidar, Saurabh Kumar Sharma, Magda H. Abdellattif, Muthupandian Saravanan, Paulraj Rajamani

**Affiliations:** ^1^School of Environmental Sciences, Jawaharlal Nehru University, New Delhi 110067, India; ^2^School of Physical Sciences, Jawaharlal Nehru University, New Delhi 110067, India; ^3^School of Computational and Integrative Sciences, Jawaharlal Nehru University, New Delhi 110067, India; ^4^Department of Chemistry, College of Science, Taif University, Al-Haweiah, P. O. Box 11099, Taif 21944, Saudi Arabia; ^5^Department of Medical Microbiology and Immunology, Division of Biomedical Sciences, School of Medicine, College of Health Sciences, Mekelle University, Tigray, Ethiopia; ^6^AMR and Nanomedicine Laboratory, Department of Pharmacology, Saveetha Dental College, Saveetha Institute of Medical and Technical Sciences (SIMATS), Chennai 600 077, Chennai, India

## Abstract

Organometallic nanoconjugates have raised great interest due to their bimodal properties and high stability. In the present study, we analyzed the cytotoxicity property of carbon dots (CDs) and a series of organometallic nanoconjugates including gold@carbon dots (Au@CDs) and silver@carbon dots (Ag@CDs) synthesized via an aqueous mode. We aimed to divulge a comparative analysis of cell proliferation, uptake, and localization of the particles in HeLa and HEK293 cell lines. Our results showed dose-dependent cytotoxicity of Au@CDs, Ag@CDs, and CDs. However, Ag@CDs showed the highest inhibition through HeLa cells with an IC_50_ value of around 50 ± 1.0 *μ*g/mL. Confocal imaging signified the uptake of the particles suggested by blue fluorescence in the interior region of HeLa cells. Furthermore, the TEM micrographs depicted that the particles are entrapped by endocytosis assisted through the cell microvilli. The CDs and Au@CDs were thus observed to be relatively safe up to a concentration of 100 *μ*g/mL and did not induce any morphological changes in the cells. Moreover, the cell proliferation assay of these nanoconjugates against HEK 293 cells signified the nontoxic nature of the nanoconjugates. The results thus revealed two major facts: firstly, the Ag@CDs had potent therapeutic potential, signifying their potential as a promising anticancer drug, and secondly, the CDs and Au@CDs at a defined dose could be used as probes for detection and also bioimaging agents.

## 1. Introduction

Engineered nanomaterials with multimodal properties have been of much focus recently, with particular emphasis on applications related to the domain of biomedicine including imaging, drug delivery, and biosensing probes [1–4]. The small size of nanoparticles (NPs) allows their easy penetration into the cells and interaction with the cellular systems [5]. Additionally, the intriguing physicochemical properties of NPs such as the size, shape, surface chemistry, and surface charge play a pivotal role in their uptake by the cells [6–9]. Due to these properties, NPs have been widely analyzed for their potential in gene delivery, target-specific drug delivery, therapeutics, and tumor targeting [10–12].

In specific, metal oxide NPs are reported for their significant biological applicability [13]. There is a plethora of reports that suggest the application of silver and gold NPs in biomedicines [14–17]. Endosome-entrapped gold NPs in the size ranging from 4 to 6 nm have been reported for their excellent uptake and bioimaging potential by HeLa and MCF-7 cell lines [15]. Carbon dots (CDs) are novel zero-dimensional carbon-based nanomaterials with relatively strong fluorescence characteristics. There has been a tremendous rise in the use of carbon dots (CDs) as fluorescent probes for bioimaging applications [18]. The synthesis methods for CD production include techniques such as laser irradiation, electrochemical oxidation, strong acid oxidation, and ultrasonic synthesis [19–22]. But, these methods suffer from disadvantages of aqueous dispersibility, expensiveness, hazardous precursors, and complex instrumentation, thereby limiting their usage in biomedicines. Therefore, researchers are now focusing on the synthesis of nanoconjugates that present the advantages of multifunctionality, targeted functionality, and superior physicochemical properties.

Regardless of the significant advances in the arena of nanotechnology, not much is understood about their cellular uptake and the subsequent mode of action. Furthermore, most of the studies nowhere suggest the toxicity assessment of these specific particles. A huge number of factors such as the dose, distribution, period of treatment, and interaction with specific biomolecule affect NP-based cellular response [23, 24]. In general, NPs are internalized by endocytic pathways wherein the uptake efficiency and resultant toxicity are correlated with the route of administration. In addition to the size and shape of the particles, charge density, cell type, stage of differentiation, and surface chemistry of NPs determine the uptake route [10, 25–27].

The uncertainties of a mode of action and compatibility before deciding its bioapplication are associated with the introduction of any new composite nanomaterial. To ensure the efficacious and harmless implementation of nanomaterials, it is essential to completely elucidate the cellular response to the nanomaterial. To eliminate the risk of toxicity and undesired *in vitro* cellular response, many parameters (cell viability, dose of particles, cell type, number of internalized particles, and degradation product) require investigation. Our prior studies report the synthesis of CDs from biocompatible precursors wherein the synthesized particles offer the advantages of aqueous solubility, stability, and high quantum yield. Additionally, we have synthesized dual-mode nanoconjugates (Au@CDs and Ag@CDs) with both well-defined optical and fluorescent properties, thereby presenting promising usage in bioimaging.

Therefore, in the present study, we coveted to investigate the toxic effects of the synthesized nanoconjugates (CDs, Ag@CDs, and Au@CDs) and understand the antiproliferation, cellular uptake, and internalization pathway. The cellular uptake and distribution of the Ag@CDs, Au@CDs, and CDs were analyzed in HeLa cell lines. Overall, our study established the cellular response of HeLa cells on exposure to CD-based nanoconjugates, fluorescence imaging potential, and intracellular uptake efficiency.

## 2. Materials and Methods

### 2.1. Synthesis of Carbon Dots (CDs)

CDs were synthesized as per our previous study [28]. PEG and citric acid were used as the precursors, and synthesis was performed via microwave-assisted method.

### 2.2. Synthesis of Au@CD/Ag@CD Nanoconjugates

The synthesized CDs were appropriately diluted and used for gold@carbon dots (Au@CDs) and silver@carbon dots (Ag@CDs) synthesis. Au@CDs were synthesized at HAuCl_4_ concentration of 0.12 mg/mL as per the protocol adopted by [28, 29]. UV-visible and fluorescence spectral analysis was performed to ascertain the synthesis of the nanoconjugates. Average particle size and morphology were determined by dynamic light scattering (DLS) and JEOL 2100F transmission electron microscope (TEM) operating at a voltage of 200 kV. The hydrodynamic size (*R*_*h*_) of particles was determined using the Stokes-Einstein equation [30] from the DLS data.

### 2.3. *In Vitro* Toxicity

#### 2.3.1. Cell Culture

The human cervical cancer cell line (HeLa) and human healthy embryonic kidney cell line (HEK293) were procured from National Centre for Cell Science, Department of Biotechnology, Pune, India. The cells were cultured in RPMI-1640 medium supplemented with 10% (*v*/*v*) FBS and antibiotics (streptomycin 10 *μ*g/mL and penicillin 100 U/mL).

#### 2.3.2. Cytotoxicity Assay

The cytotoxicity of the nanoconjugates and CDs was determined by MTT assay against HeLa and HEK293 cell lines. Briefly, 5 × 10^3^ cells/well were seeded in a 96-well plate and incubated for 24 h in an incubator maintained at 37°C and 5% CO_2_. The old media were replaced with a fresh medium containing various concentrations of nanoconjugates and CDs and incubated further for another 24 hours. Thereafter, 30 *μ*L of 1 mg/mL MTT (3-(4,5-dimethylthiazol-2-yl)-2,5-diphenyl tetrazolium bromide) and 70 *μ*L of the media were added to each well. After 4 h of incubation, the media were replenished with 100 *μ*L DMSO and incubated for 10 minutes. Absorbance was recorded in ELISA plate reader at 570 nm, and % viability was calculated as per the below-mentioned formula.

Cell Viability (%) = Mean of absorbance (Treated samples/Untreated samples)∗100.

#### 2.3.3. Determination of Reactive Oxygen Species (ROS)

Intracellular ROS generated by incubating the cell lines with nanoconjugates was estimated by 2′,7′dichlorofluorescein-diacetate (DCFHDA) staining. The cell line was seeded at a density of 5 × 10^3^ cells/well and incubated overnight at 37°C at 5% CO_2_. The cells were then treated with varying concentrations of the nanoconjugates and carbon dots and left for exposure for 24 h. The cells were thereafter washed with phosphate-buffered saline (PBS), and 40 *μ*M DCFHDA was added to each well and incubated for 30 min at 37°C. The cells were then washed twice with PBS, and fluorescence intensity was measured using 485 excitation and 520 nm emission filters using a fluorimeter (RF-5301 PC Shimadzu spectrofluorometer Nakagyo-Ku, Kyoto, Japan).

Furthermore, the ROS generated on the treatment of HeLa cell line with nanoconjugates was determined by fluorescence imaging using DCFHDA dye. The HeLa cells at 5 × 10^5^ cells/well were seeded over coverslip in six-well plates. The plate was incubated overnight that allows growth and attachment of cells. The nanoconjugates at varying concentration was added to the wells and incubated overnight at 37°C and 5% CO_2_. The cells were washed with PBS and 40 *μ*M DCFHDA and incubated for 30 min. After incubation, DCFHDA was removed, and the coverslips were suspended in PBS. The coverslips were removed and visualized on a Nikon Eclipse Ti-E (Tokyo, Japan) fluorescence microscope at 20x magnification.

#### 2.3.4. Analysis of Cell Morphology

The changes in cellular morphology of HeLa cells after treatment with nanoconjugates were analyzed using phase-contrast microscope. The cells were treated for 18 h, and any morphological variations were observed using a microscope (Nikon Eclipse Ti-S, Tokyo, Japan).

#### 2.3.5. Cellular Uptake and Bioimaging

To analyze the uptake of the nanoconjugates by the HeLa cells, the cells were treated with 50 *μ*g/mL of nanoconjugates and incubated for 6 h. After completion of the incubation period, the cells were trypsinized and centrifuged at 5000 rpm for 5 min. The pellet obtained was washed and dissolved in 1 mL of 0.1 M PBS. Imaging was performed using an Olympus FluoView TM FV1000 laser confocal microscope.

#### 2.3.6. TEM Analysis

Subcellular localization of nanoconjugates in the HeLa cells was analyzed by treating the cells with nanoconjugates and subsequent incubation for 24 hours. The treated cells were trypsinized and fixed with 2.5% glutaraldehyde for 45 min, postfixed using 1% osmic acid, and 0.1 M PBS was added to it. The cells were then dehydrated in ethanol, embedded in Epon 812, and sectioning was done using an ultramicrotome (Leica Ultracut-UCT). The sections were observed under a JEOL-JEM-2100F transmission electron microscope at 200 kV after staining with uranyl acetate.

## 3. Results

### 3.1. Characterization of Nanoconjugates

**Figure 1:**
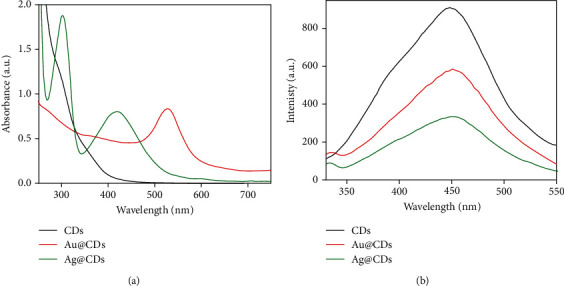
(a) UV-visible absorption spectra and (b) fluorescence spectra of synthesized CDs and nanoconjugates (Au@CDs and Ag@CDs).

The CDs synthesized using PEG and citric acid were used as the reducing agent for subsequent synthesis of Au- and Ag-based nanoconjugates. A change in color from initial yellow to purple and reddish brown provided initial evidence of Au@CD and Ag@CD formation, respectively. An evident surface plasmon resonance (SPR) peak was observed at around 530 and 420 nm for Au@CDs and Ag@CDs, respectively, in the UV-visible absorption spectra. Additionally, quenching of fluorescence intensity confirmed the formation of nanoconjugates (Figures 1(a) and 1(b)).

The detailed physical characterization (TEM, HRTEM, and EDX spectra) and mechanism of CDs and Au@CD and Ag@CD synthesis have been already published, and the data is available there in our previous study with multimode sensing application [28, 29], and hence, in this study, we explored the cell proliferation, uptake, and localization in cancerous and normal cells. The crystalline nature of the particles was confirmed from XRD analysis.  Table 1 summarizes the physical parameters of the synthesized nanoconjugates and CDs.

### 3.2. Assessment of *In Vitro* Toxicity of Synthesized Nanoconjugates

**Figure 2:**
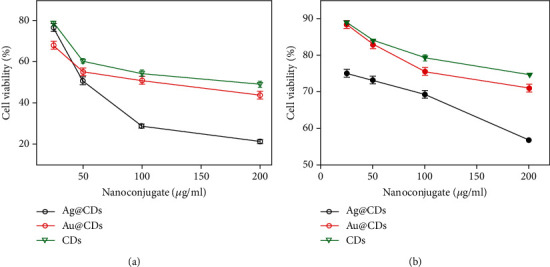
MTT assay: graph depicting the cell viability percentage as a function of varying nanoconjugate concentration: (a) HeLa and (b) HEK 293.

The antiproliferative effect of the synthesized nanoconjugates and CDs was investigated against HeLa cells via MTT assay. The cells were treated with the nanoconjugates in the concentration range of 25 to 200 *μ*g/mL for 24 hours (Figure 2). Among the particles, Ag@CDs showed the highest inhibition of HeLa cells with an IC_50_ value (concentration where 50% cell death is observed) of around 50 ± 1.0 *μ*g/mL, while CDs showed the least toxicity with an IC_50_ value of around 180 ± 0.5 *μ*g/mL. Au@CDs showed an IC_50_ value of 150 ± 0.08 *μ*g/mL, thus signifying minimal toxicity of Au@CDs. The cytotoxicity studies indicated the antiproliferative effect of the Ag@CDs in a dose-dependent manner. The literature suggests the superior toxicity of silver NPs, with an almost 60% decrease in cell viability at a mere concentration of 65 *μ*g/mL in L929 cells [31]. In the present case, the highest inactivation of cell proliferation was observed in Ag@CDs, which can be attributed to the Ag^+^ ions that form the integral core of the particles. In a similar instance, gold NPs have been reported to inhibit the proliferation of dalton lymphoma cells, with around 40-50% viability at a concentration range of 80–100 *μ*g [32]. In this study, the least toxicity of Au@CDs was observed which might be due to the CD shell over the gold particles that renders them nontoxic. Comparatively, slightly higher toxicity of Au@CDs compared to CDs can be postulated to be the formation of efficient bonding between the Au ions and the cellular surface that allows superior interaction and penetration into the cells.

**Figure 3:**
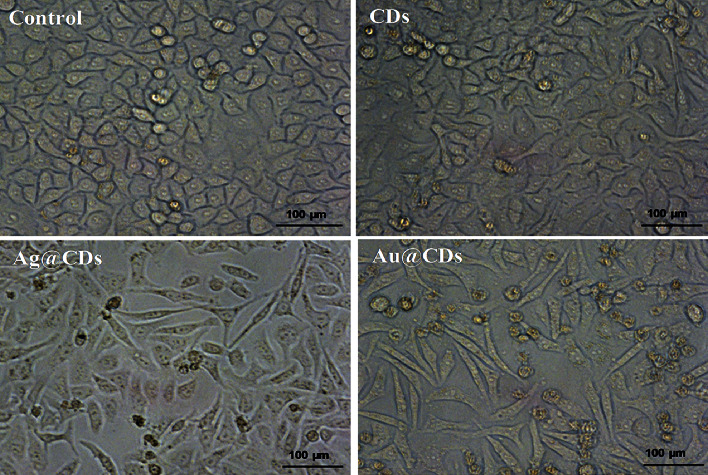
Morphological changes in HeLa cells after treatment with the nanoconjugates (100 *μ*g/mL). No morphological alterations were found in cells treated with CDs and control set. Images were captured at 20x magnification.

To observe the morphological changes induced by the nanoconjugates, images of the treated cells were taken under a microscope. The HeLa cells were treated with the nanoconjugates at 100 *μ*g/mL concentration. Distinct changes in the morphology as well as in the cell density were found compared to the control cells (Figure 3). While the control set (untreated HeLa cells) showed intact morphological features, the cells treated with Ag@CDs showed disrupted cell organization, cell shrinkage, and round cells. The cells appeared to shrink, were irregular in shape, and became round in shape. The dead cells or cells under stress showed a round morphology and get detached from the surface. Additionally, marked reductions in the number of surviving cells suggested the high toxicity and induction of apoptosis and necrosis at 100 *μ*g/mL Ag@CD concentrations. Furthermore, cells treated with CDs appeared similar in morphology to that of the control cells suggesting the nontoxicity of CDs towards HeLa cells. Likewise, the cells treated with Au@CDs showed few round cells with intact morphology signifying their lesser toxicity in comparison to Ag@CDs.

**Figure 4:**
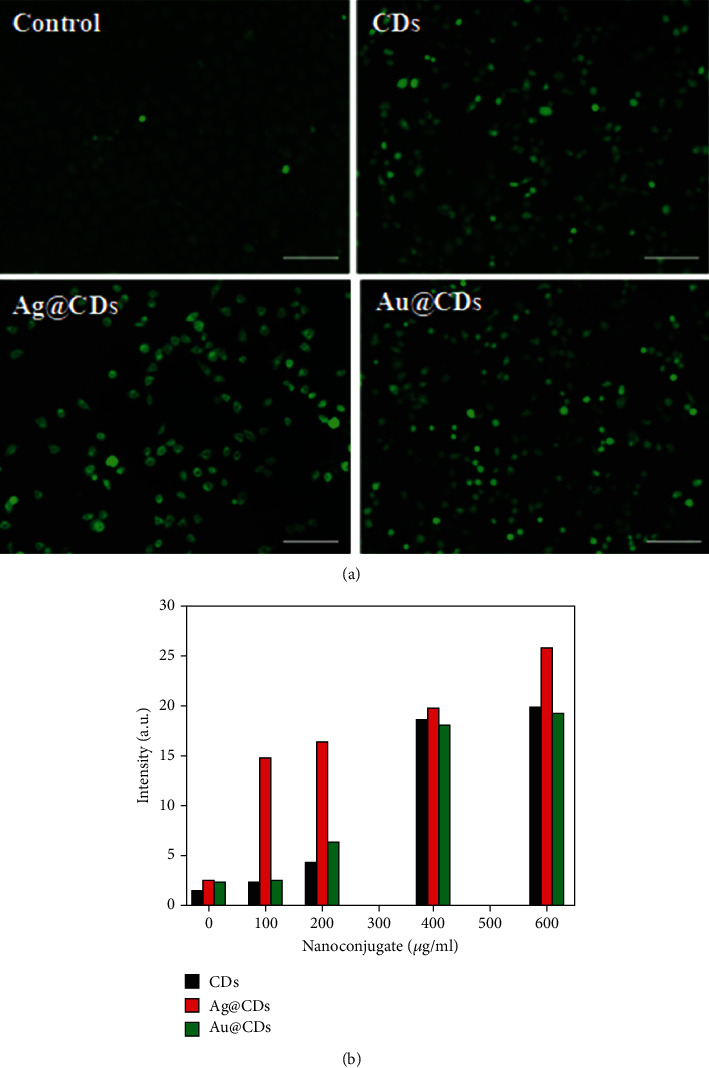
(a) Fluorescence microscopic images of DCFDA-stained cells including control cells, CD-treated cells, Ag@CD-treated cells, and Au@CD-treated cells. (b) ROS level in treated cells after incubation with the nanoconjugates as estimated by DCFDA.

The literature suggests that the induction of toxicity by NPs is generally mediated by apoptosis, mitochondrial damage, metabolic inactivity, and oxidative stress [33–36]. These processes are assisted by the production of ROS. In this study, we investigated ROS production in the HeLa cells after treatment with nanoconjugates. We used the fluorescent dye DCFHDA for analysis of ROS generation, wherein a direct correlation between the ROS amount and green fluorescence intensity is found. We did not observe any fluorescence in the control cells, while the HeLa cells treated with CDs showed weakly and diffused green fluorescence. However, the HeLa cells treated with Ag@CDs showed a high intensity of green fluorescence. Simultaneously, a decrease in the number of cells was found which suggested cell death due to high toxicity. ROS analysis thus stated that Au@CDs were less toxic compared to Ag@CDs, signified by comparatively lower fluorescence intensity (Figure 4(a)). Likewise, quantitative analysis of ROS estimation showed a relatively high intensity of DCF in Ag@CD-treated cells compared to CDs and Au@CDs (Figure 4(b)).

### 3.3. Cellular Uptake and Internalization of Nanoconjugates

**Figure 5:**
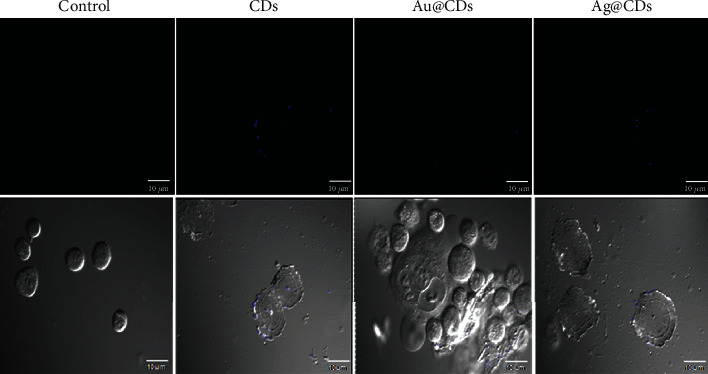
Representative confocal images of nanoconjugates treated HeLa cells after 6 h of incubation. The first set represents the scattering images, and the second set is the corresponding merged images, after incubation with CDs, Au@CDs, and Ag@CDs.

The uptake of nanoconjugates is important for analyzing the internalization of particles in cells. To analyze the potential of the synthesized nanoconjugates in live cell imaging studies and other biomedical applications, the uptake of CDs, Ag@CDs, and Au@CDs was assessed by treating the cells with 50 *μ*g/mL nanoconjugate concentrations, which was almost half the concentration that was observed to be toxic to cells.  Figure 5 shows the confocal imaging data signifying the cellular uptake of the nanoconjugates. The blue florescence signified the internalization of nanoconjugates inside the HeLa cells. The cells without any nanoconjugates treatment were taken as control for adjusting the detector gain and baseline correction. The images (Figure 5) suggest that the scattering intensity is highest for Au@CD-treated particles. All the three particles were internalized in the cells, signifying the ability of the particles to attach and be taken up by the cells. The fluorescence intensity was maximum for Au@CDs followed by Ag@CDs and CDs, respectively.

**Figure 6:**
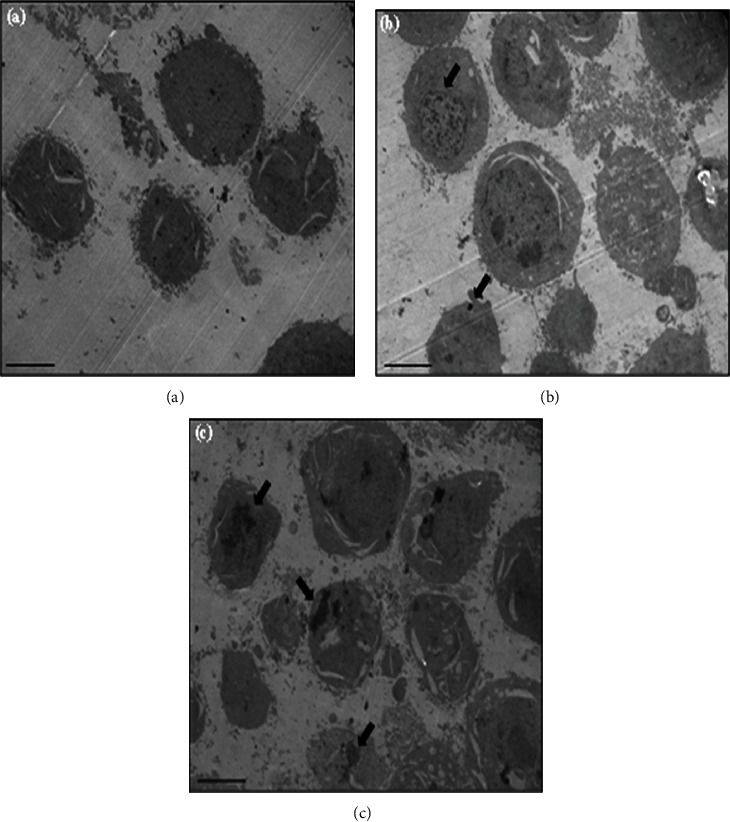
Low magnification TEM images showing intracellular localization of particles in HeLa cells. (a) untreated cells, (b) CD-treated cells, and (c) Au@CD-treated cells. The arrows in (c) and (b) show electron-dense particles corresponding to CDs and Au@CDs, respectively. Scale bar corresponds to 2 *μ*m.

The TEM further confirmed the cellular internalization of particles. The uptake of CDs and Au@CDs was studied by TEM. Due to the high toxicity of Ag@CDs, it was not possible to perform the TEM study. Due to the rapid death and subsequent detachment of cells from the adhered surface, the cell pellet could not be obtained.  Figure 6(a) shows the representative TEM images of control, CD-treated, and Au@CD-treated HeLa cells. In untreated HeLa cells, no vivid morphological changes were found, and the cell membrane was intact with an almost homogeneous cytoplasm and uniform vesicles. However, in the treated group of cells, dense aggregates were observed within the vesicles that correspond to the internalized particles (Figures 6(b) and 6(c)).

**Figure 7:**
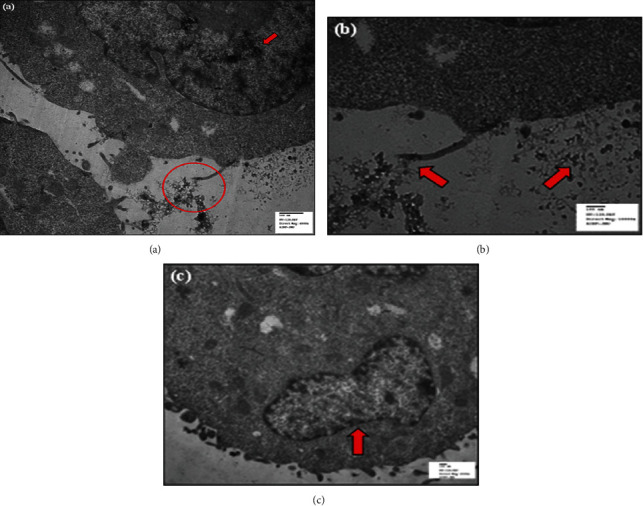
TEM images of HeLa cells treated with CDs. Images (a) and (b) correspond to the different magnification of the same cell showing the interaction of CDs with the cell microvilli. Image (c) represents the internalization of particles within cytoplasmic vesicles.

**Figure 8:**
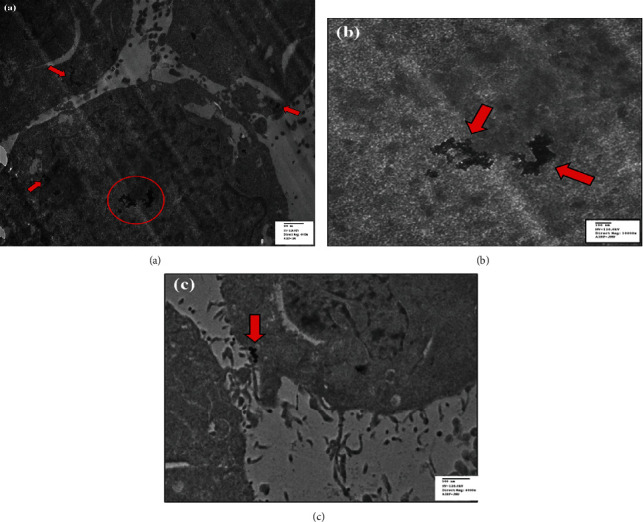
TEM images of HeLa cells treated with Au@CDs. Image (a) shows the localization of nanoconjugates within the cells. The arrows indicate the localization of Au@CDs, while image (b) corresponds to the magnified section of the same cell. Image (c) shows the interaction of particle at cell surface.

In specific, both the particles are internalized by the HeLa cells, signified by the electron-dense aggregates, shown by the red arrows in Figures 7 and 8. In the HeLa cells treated with CDs, the vesicles were quite large and were filled with ample CD aggregates. Simultaneously, the endocytotic vesicles were small and restricted to the cytoplasm, as indicated by the red arrows in Figures 7(a) and 7(c).

Additionally, as encircled in Figure 8(a), we observed the attachment of CDs to cell microvilli. The CD attachment to the microvilli has been shown by an enlarged image in Figure 7(b). A large number of aggregated particles were observed attached to the external cell surface or the plasma membrane. On the other hand, the red-encircled site in Figure 8(a) represents the localization of Au@CDs in HeLa cells. The magnified image has been shown in Figure 8(b). Notably, Figure 8(c) shows the interaction of particles on cell surface interlacing between the microvilli and cytoplasm. The images thus suggested that the particles enter the cells via endocytosis assisted by the cell microvilli. Comparative analysis suggested significantly more internalization of CDs into the cellular vesicles compared to Au@CDs, which was because of the minute size of CDs that favor its uptake both by endocytosis and diffusion through the cell surface. However, both the particle type (CDs and Au@CDs) did not induce any change in cell morphology which was consistent with the cell viability data.

## 4. Discussion

In this study, a new series of organometallic nanoconjugates including Au@CDs, Ag@CDs, and CDs were characterized by UV-visible spectroscopy, fluorescence spectroscopy, TEM, SEM, and DLS and assessed for their antiproliferative action on the HeLa cells. Fluorescence analysis signified the high quantum yield of CDs. Additionally, the synthesized Ag@CDs and Au@CDs exhibited the dual properties of optical as well as fluorescence detection [28]. Recently, quantum dots (QDs) specifically Cd-based QDs are reported for their use as *in vivo* contrast agents; however, the high toxicity offered by leaching of Cd^2+^ ions into the solution limits their applications. With regard to this, the in-house synthesized nanoconjugates have superior properties (solubility, stability, and surface accessibility) that make them promising candidates for *in vivo* applications. Cytotoxicity, in particular for Ag, Au, and CD has already been described in different cell types [37, 38]. In contrast, we analyzed the toxicity of novel dual property nanomaterials (optical and fluorescence). The aqueous solubility, easy accessibility, high stability, and quantum yield efficiency make such dual-mode nanoconjugates of superior interest in biomedical applications. Therefore, assessing the biocompatibility of these nanoconjugates is essential for determining the subsequent applications. The toxicity of nanomaterials depends on many factors such as rate of cellular uptake, particle size, and cell type [39, 40]. Cell viability assay suggested that of the three particles, Ag@CDs significantly inhibited the growth of the HeLa cells via dose-dependent manner. Most of the mechanisms postulate the production of ROS to be one of the major factors contributing towards nanomaterial-induced cell toxicity. The substrate used for synthesis as well as the nature of surface modification plays an integral role in the uptake of particles and corresponding toxicity. Consequently, we studied the generation of intracellular ROS in the HeLa cells as a response to internalized particles, using the fluorescent probe DCFDA. Indeed, DCFDA in general measures the hydroxyl, peroxyl, and other reactive oxygen species within the cell. The Ag@CD-treated HeLa cells showed relatively high ROS intensity and vivid changes in cellular morphology; apoptotic and necrotic cells were observed. The probable leaching of Ag^+^ ions into the solution and subsequent binding to the thiol groups of the inner mitochondrial membrane results in the weakening of the antioxidant defense mechanism leading to ROS formation. Accumulation of ROS results in mitochondrial disruption and release of Cyt C that in turn activates caspases ultimately resulting in cell death or DNA fragmentation [37, 38, 41]. On the other hand, CDs and Au@CDs were found to be relatively nontoxic up to a high concentration of 100 *μ*g/mL and did not induce any morphological changes. The cellular uptake and internalization of CD and Au@CDs were studied by confocal imaging and TEM. The analysis suggested that both the particles had a similar internalization process assisted by cell microvilli; however, the intracellular distribution was different. Due to the small size of CDs, they easily penetrated the cells by diffusion and were extensively accumulated within the cytosolic vesicles, while Au@CDs were mostly localized in the cytoplasmic space. It thus implies that the cellular uptake of NPs depends on the nature of material, size, shape, and surface charge [42]. Bioimaging studies demand the synthesis of nanomaterials that can easily penetrate the cells, without affecting their morphology and inducing cell death. The particles that are easily internalized and evenly distributed within the cell serve as suitable drug delivery and fluorescent markers. The results thus signify that the CDs and Au@CDs synthesized by the current protocol may serve as superior probes for biomedical and theranostic applications.

## 5. Conclusion

The Ag@CDs (50 *μ*g/mL) were found to be toxic to the HeLa cells compared to CDs and Au@CDs, thus signifying particle-type-specific toxicity. While the CD and Au@CD particles did not exhibit acute toxicity even at a high dose (100 *μ*g/mL), distinct interaction with the HeLa cells was observed. Both confocal and TEM analysis demonstrated the uptake and subsequent internalization of these particles within the cytoplasmic space and vesicles. This thus suggested that CDs and Au@CDs could be taken up by cells without any toxic effect or induction of morphological changes. Furthermore, Ag@CDs induced apoptosis in HeLa cells probably through ROS-mediated apoptotic pathway. In summary, the study divulges that cytotoxicity depends on particle composition as well as surface modification. Simultaneously, CDs and Au@CDs due to their aqueous solubility, nontoxicity, and fluorescence efficiency are suggested to be used for bioapplications, however with well-controlled concentration as cytotoxicity varies with particle dose.

## Figures and Tables

**Table 1:** Physical parameters of the synthesized nanoconjugates.

Sample	Maximum absorbance (nm)	Maximum emission (nm)	DLS R_h_ (nm)
CDs	305	454	13 ± 1
Au@CDs	520	454	47 ± 1
Ag@CDs	415	454	65 ± 2

## Data Availability

The data associated with the manuscript are available from the first and corresponding authors.
